# Effect of educational intervention on the appropriate use of oral antimicrobials in oral and maxillofacial surgery: a retrospective secondary data analysis

**DOI:** 10.1186/s12903-020-01367-1

**Published:** 2021-01-07

**Authors:** Junya Kusumoto, Atsushi Uda, Takeshi Kimura, Shungo Furudoi, Ryosuke Yoshii, Megumi Matsumura, Takayuki Miyara, Masaya Akashi

**Affiliations:** 1 Department of Oral and Maxillofacial Surgery, Kobe University Graduate School of Medicine, Kusunoki-cho 7-5-2, Chuo-ku, Kobe, Hyogo 650-0017 Japan; 2 Department of Infection Control and Prevention, Kobe University Hospital, Kobe, Hyogo 650-0017 Japan; 3 Department of Oral Surgery, Konan Medical Center, Kobe, Hyogo 658-0064 Japan

**Keywords:** Anti-bacterial agents, Antimicrobial resistance, Oral third-generation cephalosporin, Macrolide, Quinolone, Japan, Antibiotic stewardship, Oral-maxillofacial surgeon, Surgical site infection

## Abstract

**Background:**

In Japan, oral third-generation cephalosporins with broad-spectrum activity are commonly prescribed in the practices of dentistry and oral surgery. However, there are few reports on the appropriate use of antibiotics in the field of oral surgery. This study aimed to evaluate the appropriateness of antibiotic use before and after an educational intervention in the Department of Oral and Maxillofacial Surgery, Kobe University Hospital.

**Methods:**

The use of oral antibiotics was investigated among inpatients and outpatients before and after an educational intervention conducted by the antimicrobial stewardship team. Additionally, the frequency of surgical site infection after the surgical removal of an impacted third mandibular molar under general anesthesia and the prevalence of adverse effects of the prescribed antibiotics were comparatively evaluated between 2013 and 2018.

**Results:**

After the educational intervention, a remarkable reduction was noted in the prescription of oral third-generation cephalosporins, but increased use of penicillins was noted among outpatients. There was reduced use of macrolides and quinolones in outpatients. Although a similar trend was seen for inpatients, the use of quinolones increased in this population. Despite the change in the pattern of antibiotic prescription, inpatients who underwent mandibular third molar extraction between 2013 and 2018 did not show a significant increase in the prevalence of surgical site infections (6.2% vs. 1.8%, *p* = .336) and adverse effects of drugs (2.1% vs. 0%, *p* = .466).

**Conclusions:**

This study suggests that the judicious use of oral antibiotics is possible through conscious and habitual practice of appropriate antibiotic use. However, further investigation is required to develop measures for appropriate use of oral antibiotics.

## Background

Recently, antimicrobial resistance (AMR) has increased and attracted attention globally as a public health concern. Although antimicrobials are essential for the treatment of infectious diseases, the inappropriate use of antibacterial agents has led to an increase in AMR, worse clinical outcomes, and increased medical costs [1, 2]. An alarming report by O’Neill (2013) indicated that 0.7 million deaths per year can be attributed to AMR [3]. This is estimated to increase to 10 million deaths in a year, with a total gross domestic product loss of about 100 trillion USD if AMR is not addressed [3]. Based on this report, the World Health Organization (WHO) has adopted a global action plan against AMR and called to action all member countries to develop a national action plan [4]. In Japan, the use of antimicrobial drugs has been on the rise [5], and it has been estimated that additional medical costs due to AMR are estimated at 170 billion yen (approximately 1.7 billion USD) per year [6, 7]. In addition, the consumption of oral third-generation broad-spectrum cephalosporins, macrolides, and quinolones is more frequent in Japan than in Europe or the United States [5, 8–10]. This happens due to the Japanese tendency to value safety more than effectiveness and the strong tendency for prescription decisions to be made based on the "image" of the drug's strength. Thereby, in Japan, the National Action Plan on AMR 2016–2020 was formulated in April 2016, to reduce antimicrobial use per day per 1000 inhabitants to two-third of the level in 2013 by 2020. It also intended to reduce the use of oral cephalosporins, quinolones, and macrolides per day per 1000 inhabitants by 50% of the 2013 level by 2020 [6].

It has been suggested that the increase in β-lactamase-negative ABPC-resistant and penicillin-resistant *Streptococcus pneumoniae* was due to the inappropriate use of oral third-generation cephalosporins in Japan [6, 11, 12]. Additionally, the bioavailability of oral third-generation cephalosporins is low: cefditoren-pivoxil 17% and cefdinir 25% [10], which has been linked to the reduced curative response and selective AMR [6, 11, 12]. Therefore, the reduced prescription of oral third-generation cephalosporins is one of the core National Action Plans on AMR 2016–2020 [6].

Antibiotic prescription by dentists account for approximately 7–10% of all community prescriptions [13, 14]. General dental practitioners and oral and maxillofacial surgeons often prescribe antibiotics to prevent and treat bacteremia (e.g. infectious endocarditis) and the surgical site infection (SSI) that follows invasive treatments (e.g. tooth extraction) or odontogenic infections (e.g. pericoronitis). Since most odontogenic infection causing bacteria are anaerobic *Streptococci* [15–17], the first line of treatment are penicillins (e.g. amoxicillin) and often, not a broad-spectrum drug [18, 19]. However, unnecessary and excessive prescription of drugs (e.g. prescriptions for dry socket and pulpitis, and prescriptions for more than seven days routinely) have consistently been reported in the dental community [20, 21]. Additionally, surveillance reports from Japan indicate that most prescriptions of oral third-generation cephalosporins are by general dental practitioners, and this constitutes inappropriate antimicrobial use [22].

This study aimed to evaluate the current appropriateness of antibiotic use before and after the formulation and implementation of the National Action Plan on AMR 2016–2020, at the Department of Oral and Maxillofacial Surgery, Kobe University Hospital. By identifying the current level of appropriateness of antibiotic use, we will be able to plan measures to improve appropriate use of antibiotics in the dental and surgical institutions in the future.

## Methods

### Subject and analysis

In the present study, we assessed the quantity of oral antibiotics used among inpatients and outpatients in the Department of Oral and Maxillofacial Surgery, Kobe University Hospital between 2013 and 2018 (before and after the formulation and implementation of the National Action Plan on AMR 2016–2020). The following oral antibiotics were investigated: third-generation cephalosporins, macrolides, quinolones, penicillins, first- and second-generation cephalosporins, lincomycin, tetracyclines, and metronidazole. The amount of oral antibiotics used was evaluated by recording the days of therapy (DOTs) per 1,000 patient-days. For outpatients, the total number of patients in each period was used as the denominator, and DOTs per 1,000 outpatients were calculated according to previous reports [23, 24]. Further, to facilitate visual comparisons, we have expressed antimicrobial use in 2013 and 2018 as the ratio of DOTs of them (the ratio is 1: “2018 = 2013”; less than 1: “2018 < 2013”; greater than 1: “2018 > 2013”). Oral antibiotics were classified according the WHO Anatomical Therapeutic Chemical classification system. Additionally, DOTs for the whole hospital were also calculated.

### Intervention

**Fig. 1:**
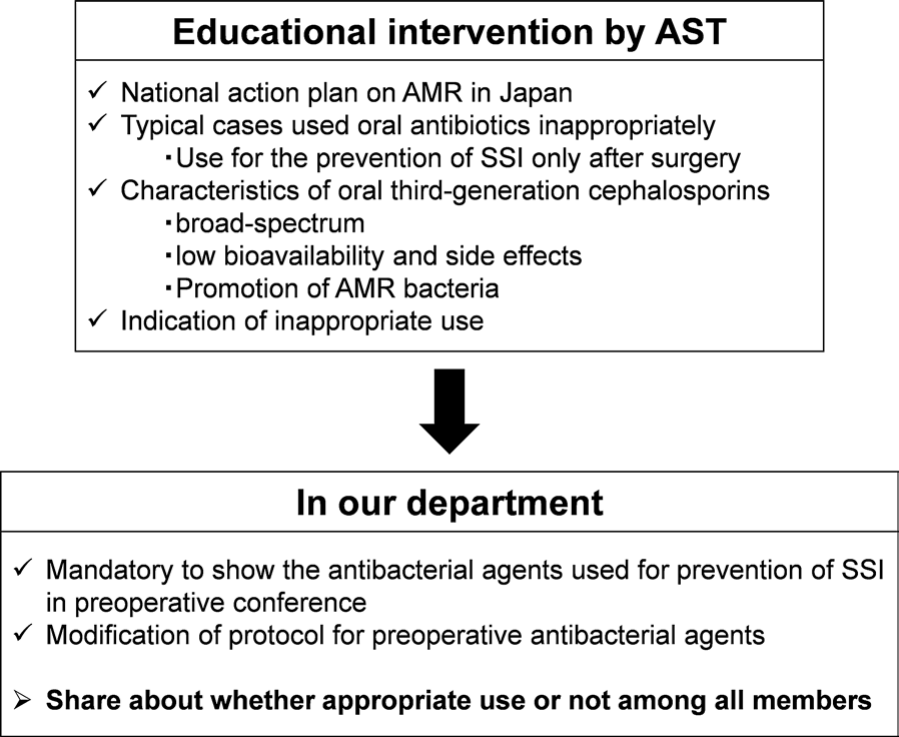
Educational intervention by the antimicrobial stewardship team and their strategy for appropriate antibiotic use. *AST* antimicrobial stewardship team, *AMR* antimicrobial resistance, *SSI* surgical site infection

Under the National Action Plan on AMR, an educational intervention by the antimicrobial stewardship team (AST) was conducted at Kobe University Hospital. The AST consisted of infectious disease physicians, pharmacists, nurses, and microbiology technologists. In addition, oral third-generation cephalosporins (cefcapene-pivoxil, cefditoren-pivoxil, cefteram-pivoxil, and cefdinir) were removed from the formulary for inpatients in our hospital since January 2018 [24]. The educational intervention included lectures for all medical staff on the appropriate use of oral antibiotics as well as educational meetings with each of the medical departments from July to August 2017 [24]. Even though the independent strategy meeting was not conducted in our department (Oral and Maxillofacial Surgery Department), the AST directives were strictly adhered to (Fig. 1).

### Clinical outcome

The prevention of infection and adverse drug effects following changes in the use of antibiotics was investigated along with an assessment of how each antibiotic was used. The frequency of SSI after the surgical removal of an impacted mandibular third molar in healthy inpatients under general anesthesia and the prevalence of adverse effects due to the prescribed antibiotics between 2013 and 2018 were comparatively analyzed. The following subjects were excluded: patients aged < 15 years, patients with immunocompromised status (e.g. diabetes mellitus, steroid, immunosuppression, hemodialysis), and patients with a prior infection in the surgical site. The protocol for use of antibacterial agents to prevent postoperative infection in our department was modified from “cefmetazole 2 g/day for 3 days + oral antibiotics for 3 days” in 2013 to “cefmetazole 2 g/day for 1 day + oral antibiotics for 1 day” in 2018 following the guidelines for the prevention of postoperative infection [25]. The diagnostic criteria for SSI after impacted third molar extraction surgery was defined with reference to the criteria for defining SSI provided by the Centers for Disease Control and Prevention (CDC) [26]. SSIs included infections occurring within 30 days of surgery (tooth extraction) involving only the extraction socket or the intraoperative tissue manipulation with at least one of the following: (1) purulent drainage from the surgical region; (2) organism isolation from the surgical area; (3) at least one of the signs of infection (tenderness, localized swelling, redness, or heat); or (4) diagnosis of SSI by the surgeon or attending dentist.

### Statistical analyses

For comparisons between the two groups in 2013 and 2018, DOTs with third-generation cephalosporins, macrolides, quinolones, and penicillins were calculated using monthly data aggregating each antibiotic use. For comparisons between the two groups in 2013 and 2018 in relation to impacted third molar extraction, Fisher’s exact test was used for nominal variables and the Mann–Whitney U test was used for continuous variables. The significance level was set at *p* = 0.05. R software version 3.4.1 (R Development Core Team, 2017, R Foundation for Statistical Computing, Vienna, Austria) was used for statistical analyses.

## Results

### Oral antibiotic use

**Fig. 2:**
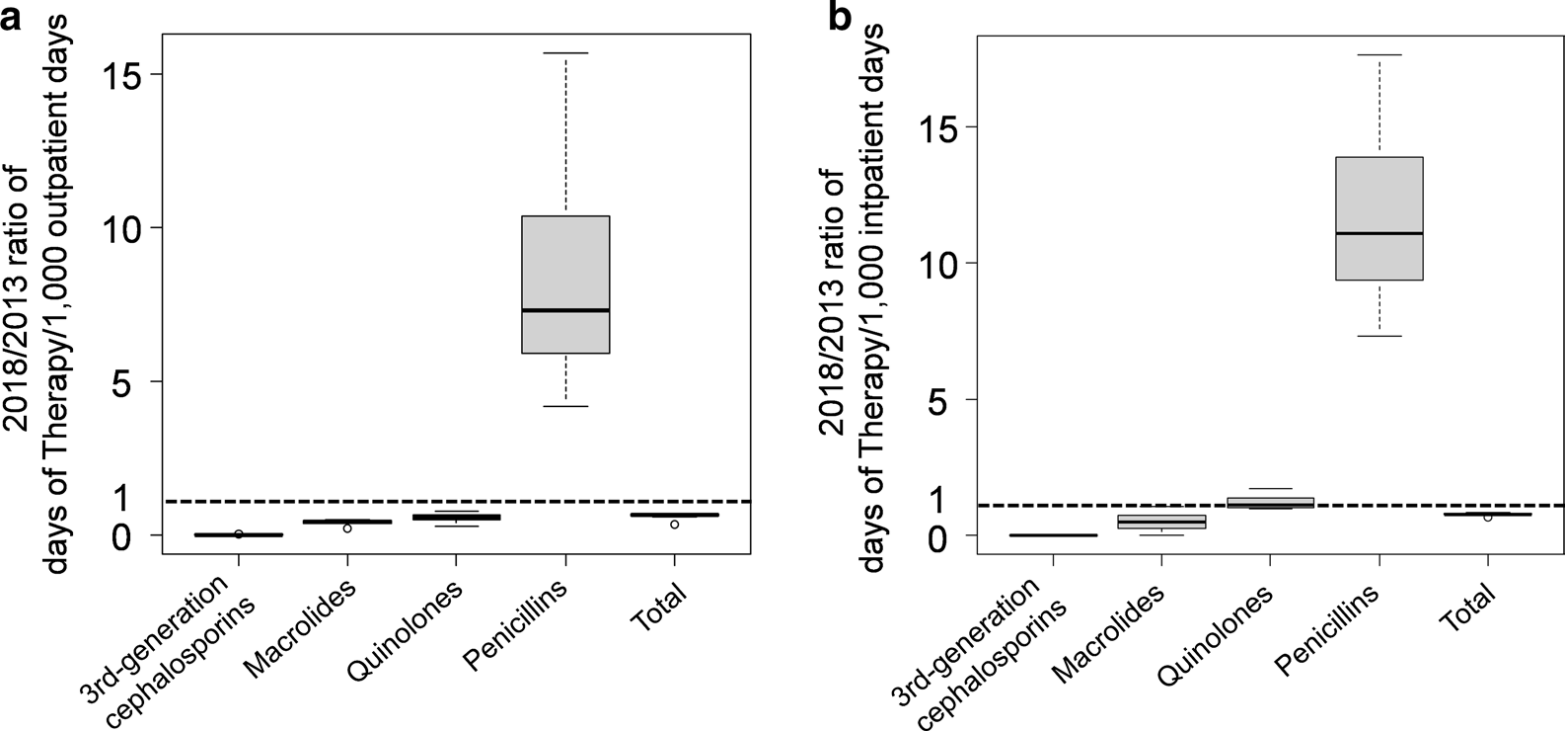
Variations in oral antibiotic use at our department. The variations show the ratio of DOTs between 2018 and 2013. The dotted lines indicate similar DOTs in 2018 and 2013. **a** Outpatients: third-generation cephalosporins (median 0.01); macrolides (median 0.4); quinolones (median 0.6); penicillin (median 7.3); and the total amount of oral antibiotics (median 0.7). **b** Inpatients: third-generation cephalosporins (median 0); macrolides (median 0.5); quinolones (median 1.1); penicillins (median 11.1); and the total amount of oral antibiotics (median 0.8). DOTs, days of therapy

The total amount of oral antibiotics used was reduced markedly in 2018 compared to that used in 2013 for both inpatients and outpatients. Moreover, the use of oral third-generation cephalosporins was greatest for both inpatients and outpatients in 2013; however, it was supplanted by penicillins in 2018 (Table 1; Additional file 1: Table S1). The most commonly used antibiotics of each type were as follows: amoxicillin in penicillins, medium-acting type in macrolides (clarithromycin), and third-generation quinolones. For outpatients, both macrolide and quinolone use decreased in 2018 compared to that in 2013 (Fig. 2a; Additional file 1: Fig. S1). For inpatients, compared to that in 2013, macrolide use decreased and quinolone use increased in 2018 (Fig. 2b; Additional file 1: Fig. S2).

**Table 1:** DOTs per 1000 patient-days of oral antibiotics in the Department of Oral and Maxillofacial Surgery

	Outpatients		Inpatients	
	2013	2018	2013	2018
First- and second-generation cephalosporins^a^	0 (0, 0)	0 (0, 0)	0 (0, 0)	0 (0, 0)
Third-generation cephalosporins^b^	218 (197, 233)	2.5 (1.4, 3.3)^i^	170 (142, 199)	0 (0, 0)^i^
Macrolides	169 (158, 179)	70.8 (60.2, 81.4)^i^	59.4 (47.6, 73.8)	28.3 (11.6, 32.9)^i^
Short-acting^c^	0 (0, 0)		0 (0, 0)	0 (0, 49.4)^j^
Medium-acting^d^	138 (125, 162)	0 (0, 0)	41.9(19.2, 49.6)	0 (0, 5.0)^i^
Long-acting^ee^	27.2 (19.5, 37.0)	52.9 (41.0, 68.7)^i^	18.8 (17.6, 25.6)	10.5 (5.5, 19.1)^i^
Quinolones	57.0 (41.7, 80.9)	15.9 (14.0, 19.4)^i^	21.4 (13.3, 32.5)	24.5 (14.6, 32.9)
Second-generation^f^	9.4 (8.1, 16.9)	35.6 (28.6, 40.8)^i^	16.6 (7.9, 32.5)	0 (0, 1.9)^i^
Third-generation^g^	47.7 (34.1, 68.8)	7.3 (4.4, 10.2)	0 (0, 0)	19.5 (14.6, 28.2)^j^
Penicillins	25.3 (18.9, 32.3)	24.9 (23.5, 32.5)^i^	7.1 (6.0, 12.7)	115 (79.8, 118)^j^
Amoxicillin	25.3 (18.9, 32.3)	185 (170, 195)^j^	7.1 (6.0, 12.7)	103 (74.7, 116)
Clavulanate/amoxicillin	0 (0, 0)	181 (168, 195)	0 (0, 0)	0 (0, 12.7)
Clindamycin	0 (0, 1.5)	0.8 (0, 7.0)	0 (0, 0)	13.4 (10.5, 19.4)^j^
Tetracyclines^h^	0 (0, 0)	2.5 (0. 5.1)	0 (0, 1.1)	0 (0, 4.6)
Metronidazole	0 (0, 0)	0 (0, 2.0)^j^	0 (0, 0)	12.6 (0, 18.9)^j^
Fosfomycin	0 (0, 0)	5.1 (0, 13.9)^j^	0 (0, 0)	0 (0, 0)
Others		0 (0, 0)		
Sulfamethoxazole/	0 (0, 0)		0 (0, 0)	0 (0, 5.1)
Trimethoprim		0 (0, 0)		
Total	464 (451, 502)	310 (282, 345)^i^	278 (227, 319)	211 (176, 250)^i^

Median (first quartile, third quartile)

*DOTs* days of therapy

^a^Cefalexin, cefaclor

^b^Cefcapene pivoxil, cefditoren–pivoxil, cefteram–pivoxil, cefdinir

^c^Erythromycin

^d^Clarithromycin, roxithromycin

^e^Azithromycin

^f^Levofloxacin, ciprofloxacin, tosufloxacin, ofloxacin

^g^Sitafloxacin, garenoxacine, moxifloxacin, prulifloxacin

^h^Minocycline, doxycycline

^i^DOTs in 2018 decreased significantly compared to those in 2013

^j^DOTs in 2018 increased significantly compared to those in 2013

### SSI and adverse effects

**Fig. 3:**
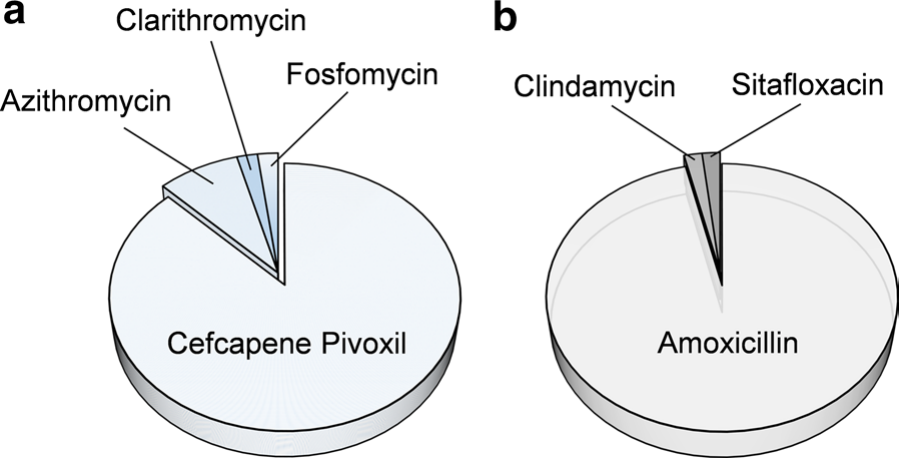
Oral antibiotics prescribed for inpatients after surgical removal of an impacted mandibular third molar. **a** In 2013, cefcapene–pivoxil was the most commonly used antibiotic (87.5%). **b** In 2018, amoxicillin was the most commonly used antibiotic (96.4%)

The oral antibiotics included in the treatment protocol at our department were cefcapene-pivoxil (87.5%), azithromycin (8.3%), clarithromycin (2.1%), and fosfomycin (2.1%) in 2013 and amoxicillin (96.4%), clindamycin (1.8%), and sitafloxacin (1.8%) in 2018 (Fig. 3).

The surgical removal of an impacted mandibular third molar was performed in 48 patients in 2013 and 55 patients in 2018. There was no significant difference in the background factors, other than the prescribing periods of oral antibiotics (Table 2). SSI occurred in 3 patients (6.2%) in 2013 and in 1 patient (1.8%) in 2018 (*p* = 0.336). Adverse effects following the administration of antibiotics were also noted in 1 patient (2.1%) in 2013 (skin rash after azithromycin administration), but no adverse effects developed in any patient in 2018 (*p* = 0.466) (Table 3). Note that, in our department, the number of cases of osteomyelitis was higher in 2018 than in 2013 (data not shown). This needs to be further assessed.

**Table 2:** Clinical characteristics of subjects who underwent impacted mandibular third molar removal in 2013 and 2018

	2013 (n = 48)	2018 (n = 55)	*p* value
Sex (male)	21 (43.8%)	22 (40%)	.841
Age	37.5 (24.8, 52)	33 (24, 55.5)	.947
Smoking	7 (14.6%)	13 (23.6%)	.320
Alcohol consumption	11 (22.9%)	14 (25.5%)	.821
Allergy	9 (18.1%)	8 (14.5%)	.604
Extraction tooth number	2 (1, 3)	2 (1, 3)	.520
Operation time (min)	81.5 (51.8, 112)	84 (58, 108)	.443
Prescription period of total antibiotics (intravenous and oral)	6 (6, 6)	2 (2, 3)	< .001
Prescription period of oral antibiotics	3 (1, 6)	1 (1, 6)	< .001

Median (first quartile, third quartile)

**Table 3:** SSI incidence after surgery and adverse effects of prescribed antibiotics

	2013 (n = 48)	2018 (n = 55)	*p* value
SSI	3 (6.2%)	1 (1.8%)	.336
Adverse effect	1 (2.1%)	0	.466

*SSI* surgical site infection

## Discussion

The following trend was observed in this study: the total amount of antibiotics used decreased; oral third-generation cephalosporins were no longer prescribed; and amoxicillin became the most commonly used medications at the Department of Oral and Maxillofacial Surgery, Kobe University Hospital. Additionally, the amount of macrolides used also decreased, and the general appropriateness of antibiotic use improved based on the action plan for AMR. Our study is relevant because thus far, only few reports, especially in the dental surgery field, have evaluated the impact of the global action plan on AMR on the appropriate use of antibiotics [27–29].

In the present study, educational interventions and conscious and habitual practice of appropriate antibiotic use could reduce the use of antimicrobials. In addition, the use of antimicrobials need not be broad-spectrum [15–17], and as narrow a range of beta-lactams (e.g. AMPC) as possible was considered preferable. The results of this study suggest that prophylactic antimicrobials should be administered in 1–2 days, but further investigation is necessary because the administration of prophylactic antimicrobials may vary depending on the target surgery. In contrast, there is no conclusion from this study about the necessity of antimicrobial administration, the type of antimicrobial agent, and the duration of treatment for dental infections due to differences in the type and degree of disease addressed. Therefore, more detailed studies are needed in the future. By actively addressing these issues, we believe that we can contribute to the proper use of antimicrobials in the field of dentistry and oral and maxillofacial surgery.

In this study, for outpatients, there was almost no prescription of oral third-generation cephalosporins in 2018. Moreover, macrolides and quinolones prescriptions in 2018 were much less than that in 2013, because of the adoption of the National Action Plan on AMR in Japan [6]. The introduction of educational intervention by the AST was considered to have contributed significantly in this regard [24]. In addition, we consider that these results reflect the high compliance following the introduction of the guidelines in Japan (e.g., guidelines for the treatment of odontogenic infection [30] or guidelines for the prevention of postoperative infection [25]). The sharing of knowledge and creation of awareness about appropriate antimicrobial agents contributed significantly to the appropriate antibiotic use observed in our department. All members of our department participated in clinical conferences where guidelines on the appropriate use of antibiotics for surgical patients were discussed in the department’s conference room (a staff member (SF) specialized in odontogenic infection who is an “infection control doctor” certified in Japan as a specialist in infection control must attend). Thus, this probably influenced our routine daily clinical practice even though a departmental antibiotic policy was not adopted. However, we still came across cases of inappropriate use of antibiotics, and we would like to investigate this further in a subsequent study.

For inpatients, there were no prescriptions of oral third-generation cephalosporins in our department in 2018. Thus, oral third-generation cephalosporins were removed from the formulary for inpatients in our hospital, and the educational intervention by the AST contributed significantly in this regard [24]. Penicillins, which are highly effective against odontogenic infection, have supplanted oral third-generation cephalosporins in terms of the appropriate use of antibiotics [18, 19]. However, the prescription of quinolones, particularly Sitafloxacin, increased in 2018 compared to that in 2013. This might have been influenced by change in disease pattern encountered in our department. The number of surgical treatments performed for inpatients with intractable osteomyelitis, medication-related osteonecrosis of the jaw (MRONJ), and osteoradionecrosis in our department increased since 2016. Hence, it was considered that the residual cases of infectious symptom had increased postoperatively, with increased postoperative use of antimicrobial agents. Owing to its good antibacterial bone penetration [31] and significant effect in treatment-resistant osteomyelitis [32], there seemed to be an increase in the use of quinolones. Sitafloxacin was found to be particularly effective for MRONJ [33]. However, this report had a low evidence level, and the results were not validated against narrower spectrum antibiotics such as penicillins (amoxicillin or clavulanate/amoxicillin). This subject should be the research agenda for future studies since there is possibility of the persistence of inappropriate use of antibiotics.

The clinical outcomes and prevalence of adverse effects with changes in antibiotic use were investigated among inpatients. We observed an SSI incidence of 6.2% in 2013 and 1.8% in 2018. In addition, the prevalence of adverse effects due to antibiotics was 1.8% in 2013, but no adverse effects were observed in 2018. It has previously been reported that the prevalence of SSI after surgery for mandibular impacted third molar extraction was around 10% [34]; postoperative SSI developed in 4% of patients in the antibiotic group and 6.1% of patients in the placebo group in a meta-analysis assessing the effectiveness of antibiotic prophylaxis [35]. Prevalence of adverse effects of antibiotics reported for penicillins and cephems was 6–7% [36]. Amoxicillin was the safest antibiotic prescribed by dentists [37]. In this study, although the sample size was small, there was not much difference in the findings when compared to past reports [34–36], and the incidence of SSI and adverse effects were also similar. These findings suggest that these negative impacts (SSI and adverse effects) exist in clinical practice despite the change in antibiotics. Although the need for prophylactic antibiotic administration for third molar extraction is controversial, its use is currently favored [38]. Presently, postoperative oral antibacterial agents are not prescribed for the prevention of SSI in our department, and further investigation needs to be undertaken to determine the influence of not prescribing oral antibacterial agents on the onset of SSI.

There were several limitations in this study. Since the investigation included data from a single department, the results do not reflect the entirety of the Japanese dental institutions. However, we consider that the results might be a useful starting point in promoting appropriate use of antibiotics. Further, it was difficult to evaluate the posology of antibiotics since “DOT” was the measure for evaluating the amount of antibiotics used in this study. It was further difficult to evaluate an appropriate posology in this study, since the dosage of antibiotics used in Japan is generally lower than the world standard (approximately 1/3–1/2 in almost antimicrobial agent according to a package insert). Especially, there are strict restrictions on antibiotics prescription and dosages in dentistry. In contrast, the defined daily dose (DDD), which is based on the total number of grams of the antimicrobial agent used, is an indicator for evaluating other antimicrobial amounts. In “DDDs,” it is not possible to determine which factors are problematic in the case of large doses, such as the daily dose, the number of days of the administration, or the number of people treated. In addition, it is difficult to determine changes in the amount of antimicrobial agents used when the dosage is changed because many Japanese medical departments (not dentistry and oral surgery) have recently been administering antimicrobial agents according to global standards. In this study, not a few hospitalized cases have been intervened by the medical doctors, and the dosage of antimicrobial agents must be reduced due to the inclusion of elderly patients and those with impaired renal function. Therefore, in the present study, we determined that DOT, without taking into account the daily dose, was appropriate for the evaluation of antimicrobial use, as per previous reports [23, 24]. Another limitation was the switch from intravenous to oral antibiotics for some patients which could have potentially altered the results. Therefore, inappropriate prescription of oral antibiotics continues to plague Japan warranting further impetus to this issue.

## Conclusions

This study suggested that the appropriate use of oral antibiotics in our department was improved following an educational intervention and through habitual practice of appropriate antibacterial use. However, further investigation is needed regarding the observed increase in the use of quinolones. Based on the results of this study, we consider that it is necessary to include dentists under the National Action Plan on AMR. There is also a need for general dental practitioners to cooperate and share data in order to ensure an effective plan to reduce inappropriate use of antibiotics.

## Supplementary information

**Additional file 1.** The amount of use of oral antibiotics in Kobe University Hospital.

## Data Availability

The datasets used and/or analyzed during the current study are available from the corresponding author on reasonable request.

## Abbreviations

AMR: Antimicrobial resistance
AST: Antimicrobial stewardship team
CDC: Centers for Disease Control and Prevention
DOTs: Days of therapy
GDP: Gross domestic product
MRONJ: Medication-related osteonecrosis of the jaw
SSI: Surgical site infection
WHO: World Health Organization
