# Supplementary material for: Effect of educational intervention on the appropriate use of oral antimicrobials in oral and maxillofacial surgery: a retrospective secondary data analysis
Source: BMC Oral Health. 2021 Jan 7;21:20. doi: 10.1186/s12903-020-01367-1 (PMC7791648; doi:10.1186/s12903-020-01367-1)
Supplement: Supplementary file 1 — Additional file 1. The amount of use of oral antibiotics in Kobe University Hospital. [file 12903_2020_1367_MOESM1_ESM.docx]

**Additional file 1**

**Effect of educational intervention on the appropriate use of oral antimicrobials in oral and maxillofacial surgery** – a retrospective secondary data analysis

Junya Kusumoto^1, 2*^, Atsushi Uda^2^, Takeshi Kimura^2^, Shungo Furudoi^1, 3^, Ryosuke Yoshii^1^, Megumi Matsumura^1^, Takayuki Miyara^2^, Masaya Akashi^1^

1. Department of Oral and Maxillofacial Surgery, Kobe University Graduate School of Medicine, Kobe, Hyogo, 650-0017, Japan

2. Department of Infection Control and Prevention, Kobe University Hospital, Kobe, Hyogo, 650-0017, Japan

3. Department of Oral Surgery, Konan Medical Center, Kobe, Hyogo, 658-0064, Japan

*Correspondence and requests for materials should be addressed to Junya Kusumoto

Department of Oral and Maxillofacial Surgery, Kobe University Graduate School of Medicine, Kusunoki-cho 7-5-2, Chuo-ku, Kobe 650-0017, Japan

Tel.: +81-078-382-6213; FAX: +81-078-382-6229

E-mail: [chivalry_2727@yahoo.co.jp](mailto:chivalry_2727@yahoo.co.jp)

**Additional file 1: Figures**


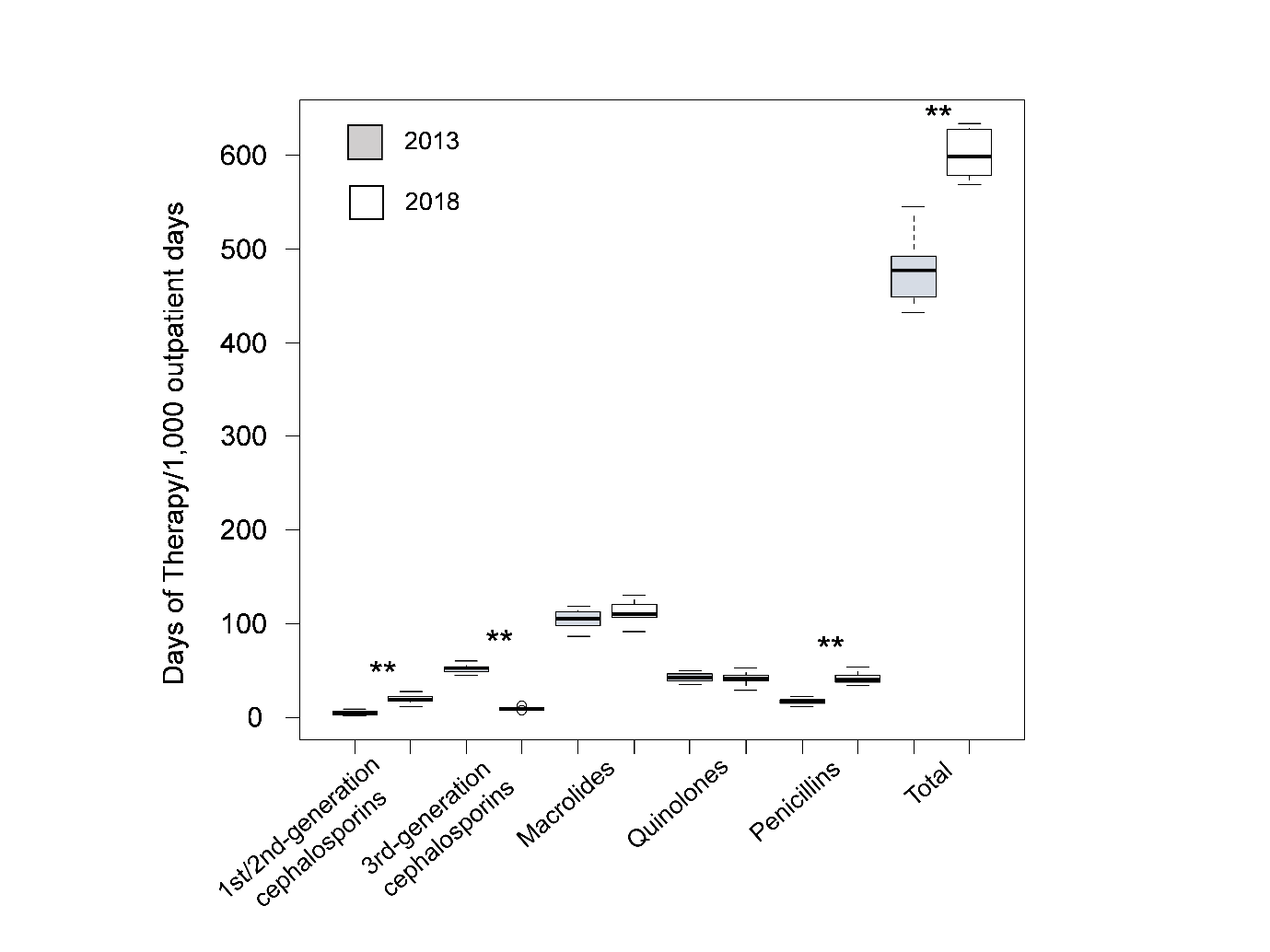


**Additional file 1: Fig. S1.** The amount of use of oral antibiotics for outpatients in Kobe University Hospital. Third-generation cephalosporins were reduced significantly in 2018 compared to in 2013 (***p* < 0.001). Macrolides and Quinolones were almost equal in 2018 compared to in 2013 (*p* = 0.143, 0.840). First and second-generation cephalosporins and Penicillins were increased significantly in 2018 compared to in 2013 (***p* < 0.001). The total amount of use of oral antibiotics was increased significantly in 2018 compared to in 2013 (***p* < 0.001).


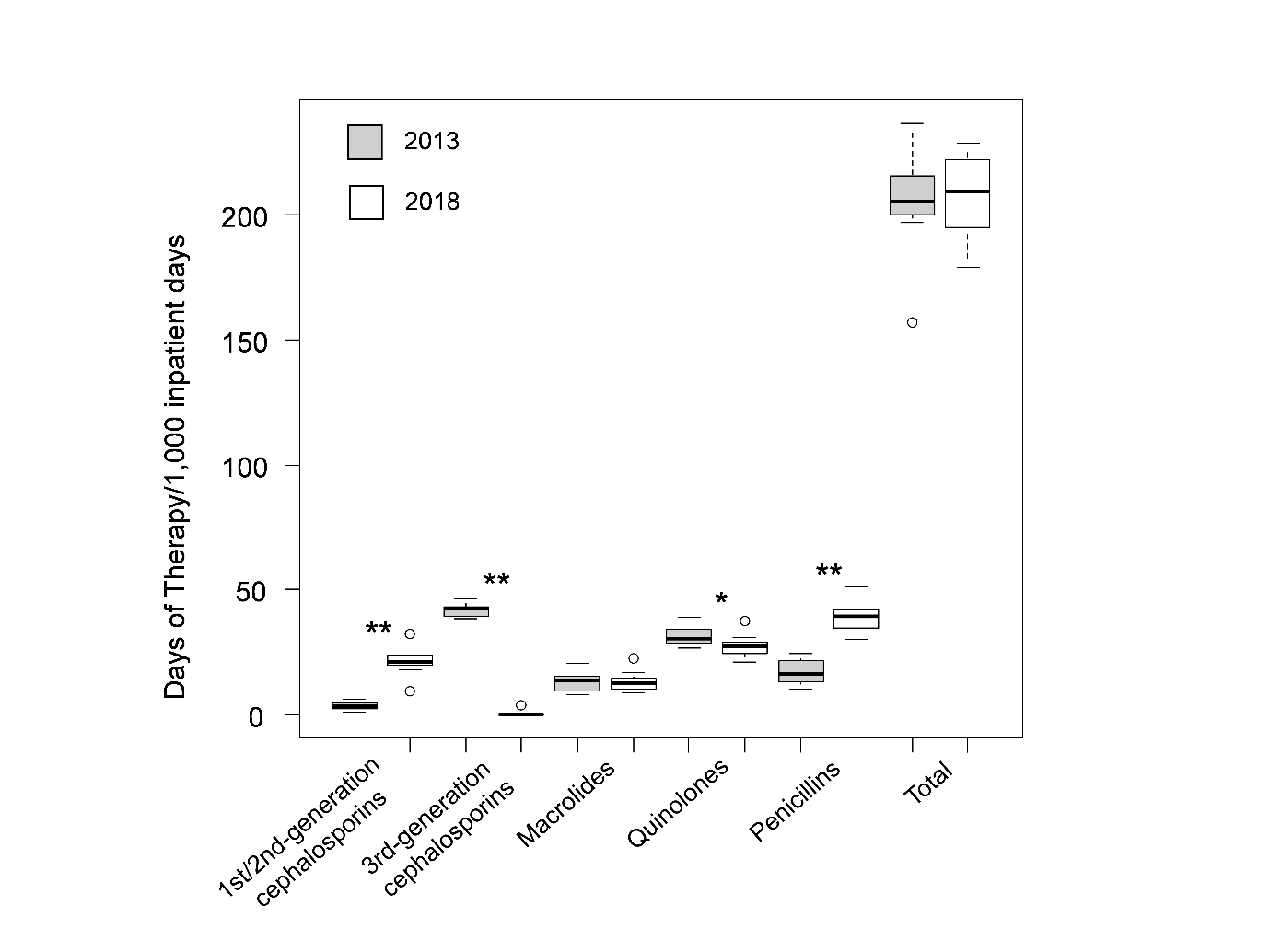


**Additional file 1: Fig. S2.** The amount of use of oral antibiotics for inpatients in Kobe University Hospital. Third-generation cephalosporins and Quinolones were reduced significantly in 2018 compared to in 2013 (***p* < 0.001, **p* = 0.018). Macrolides were almost equal in 2018 compared to in 2013 (*p* = 0.908). First and second-generation cephalosporins and Penicillins were increased significantly in 2018 compared to in 2013 (***p* < 0.001). The total amount of use of oral antibiotics was almost equal in 2018 compared to in 2013 (*p* = 0.843).

**Additional file 1: Table S1** The days of therapy per 1,000 patient days of oral antibiotics in Kobe University Hospital

|  | Outpatients | | Inpatients | |
| --- | --- | --- | --- | --- |
|  | 2013 | 2018 | 2013 | 2018 |
| First and second-generation cephalosporins^1^  Third-generation cephalosporins^2^  Macrolides  Short-acting^3^  Medium-acting^4^  Long-acting^5^  Quinolones  Second-generation^6^  Third-generation^7^  Penicillins  Amoxicillin  Clavulanate/Amoxicillin  Clindamycin  Tetracyclines^8^  Metronidazole  Fosfomycin  Others  Sulfamethoxazole/  Trimethoprim  Total | 3.8 (3.0, 5.8)  52.4 (48.7, 53.8)  105 (98.8, 111)  1.9 (1.6, 2.6)  96.8 (91.9, 105)  4.9 (4.6, 5.5)  42.1 (39.1, 46.1)  35.5 (34.2, 38.4)  6.6 (5.0, 7.4)  15.9 (15.2, 18.4)  11.2 (10.6, 12.3)  5.2 (4.7, 6.8)  2.9 (1.6, 3.7)  29.5 (28.0, 30.7)  2.3 (1.5, 3.3)  1.3 (0.9, 1.6)  221 (208, 229)  477 (450, 492) | 19.2 (16.9, 22.0)  8.9 (8.6, 9.3)  110 (107, 118)  5.0 (3.8, 5.5)  103 (95.0, 108)  2.6 (2.2, 2.8)  41.8 (38.9, 45.2)  36.2 (32.4, 38.9)  6.4 (5.1, 7.0)  40.3 (38.3, 44.7)  29.1 (28.0, 29.7)  11.5 (10.3, 15.0)  3.9 (3.3, 4.1)  49.2 (43.7, 54.6)  1.3 (0.8, 1.7)  0.2 (0.1, 0.3)  322 (306, 334)  599 (580, 627) | 3.3 (2.4, 4.5)  42.5 (39.6, 42.9)  13.4 (10.0, 15.2)  0.9 (0, 1.4)  10.0 (7.9, 13.1)  1.8 (1.5, 2.1)  30.3 (28.5, 33.5)  28.4 (27.1, 32.2)  1.6 (1.3, 2.1)  16.6 (13.1, 20.5)  9.3 (7.8, 12.3)  6.7 (5.5, 8.8)  0.8 (0.6, 1.0)  6.0 (5.3, 6.9)  5.8 (4.5, 6.5)  0.2 (0, 1.4)  86.1 (81.7, 92.7)  205 (200, 215) | 21.3 20.2, 24.0)  0 (0, 0)  12.8 (10.8, 14.4)  1.0 (0.5, 2.0)  10.9 (8.8, 12.0)  0.7 (0.4, 0.9)  27.4 (24.2, 28.5)  24.6 (23.4, 26.6)  2.8 (2.2, 3.5)  40.0 (34.8, 41.5)  25.0 (22.0, 26.0)  14.6 (13.4, 16.8)  1.1 (0.8, 0.7)  17.1 (10.7, 18.4)  1.4 (1.3, 2.3)  0 (0, 0)  89.7 (81.2, 94.7)  213 (200, 222) |

Median (first quartile, third quartile)

1. Cefalexin, Cefaclor

2. Cefcapene Pivoxil, Cefditoren Pivoxil, Cefteram Pivoxil, Cefdinir

3. Erythromycin

4. Clarithromycin, Roxithromycin

5. Azithromycin

6. Levofloxacin, Ciprofloxacin, Tosufloxacin, Ofloxacin

7. Sitafloxacin, Garenoxacine, Moxifloxacin, Prulifloxacin

8. Minocycline, Doxycycline
